# World Hepatitis day 2021 –screening and vaccination against Hepatitis B virus in Accra, Ghana

**DOI:** 10.1186/s12889-023-16108-6

**Published:** 2023-06-16

**Authors:** Kwadwo Asamoah Kusi, William van der Puije, Diana A. Asandem, Rawdat Baba-Adam, Hardy Agbevey, Bright Asare, Philip Segbefia, Lutterodt Bentum-Ennin, Audrey Annan, Frank Osei, Doreen Teye-Adjei, Elsie Sutaya Galevo, Gifty Odame, Gloria Ansa, Linda Amoah, Joseph Humphrey Kofi Bonney

**Affiliations:** 1 Department of Immunology, Noguchi Memorial Institute for Medical Research, University of Ghana, Legon Accra, Ghana; 2 Department of Biochemistry, Cell and Molecular Biology, University of Ghana, Legon Accra, Ghana; 3 Department of Virology, Noguchi Memorial Noguchi Memorial Institute for Medical Research, University of Ghana, Legon Accra, Ghana; 4 Department of Animal Biology and Conservation Science, University of Ghana, Legon Accra, Ghana; 5 Department of Theoretical Medicine, Kwame Nkrumah University of Science and Technology, Kumasi, Ghana; 6 University Hospital, University of Ghana, Legon Accra, Ghana

**Keywords:** World Hepatitis Day, Viral Hepatitis, HBV vaccinations, Hepatitis-Malaria (HEPMAL), Awareness

## Abstract

**Background:**

In Ghana, Hepatitis B virus (HBV) infection remains a major public health threat as in many parts of the world. Even with an effective vaccine, there are shortfalls with low vaccine coverage among adults. To create awareness and encourage vaccination, community engagement and public-private partnerships are needed in endemic settings to help fund campaigns and offer screening and vaccinations at no cost to under privileged people.

**Objectives:**

An awareness and screening exercise was scheduled by University of Ghana-based Hepatitis-Malaria (HEPMAL) project team to coincide with the World Hepatitis Day (WHD) 2021. It was to engage the community in creating awareness of the menace and offer diagnostic services to ascertain prevalence levels and provide needed clinical support.

**Methods:**

Participants from the University of Ghana community and its immediate environs were registered, taken through pre-counselling sessions where they were educated on hepatitis transmission and prevention before consenting. Eligible participants were screened for HBV markers (HBsAg, HBeAg, HBsAb, HBcAb,HbcAg) with a rapid test kit. All HBsAb-negative participants were recommended for initial vaccination at the event, whilst the subsequent shots were administered at the University Hospital Public Health Department. Hepatitis B surface Antigen-positive participants were counselled and referred for appropriate care.

**Results:**

/ **Outcomes**: A total of 297 people, comprising of 126 (42%) males and 171 (58%) females aged between 17 and 67 years were screened during the exercise. Amongst these, 246 (82.8%) showed no detectable protective antibodies against HBV and all of them agreed to and were given the first dose HBV vaccine. Additionally, 19 (6.4%) individuals tested positive for HBsAg and were counselled and referred to specialists from the University Hospital for further assessment and management. We found that 59 (19.9%) of our participants had previously initiated HBV vaccination and had taken at least one dose of the vaccine more than 6 months prior to this screening, 3 of whom tested positive for HBsAg. For the three-dose HBV vaccines deployed, a little over 20% (50/246) and a further 17% (33/196) did not return for the second and the third doses respectively, resulting in an overall 66% (163/246) of persons who completed all three vaccinations.

**Conclusions:**

/ **Lessons learnt**: Our medical campaign exercise established an active case prevalence rate of 6.4% and achieved a full vaccination success rate of 66% which is critical in the induction of long-term immunity in the participants. Aside these achievements, we would like to reiterate the importance of the use of different approaches including educational events and WHD activities to target groups and communities to raise awareness. Additionally, home and school vaccination programmes may be adopted to enhance vaccine uptake and adherence to the vaccination schedule. We plan to extend this screening exercise to deprived and/or rural communities where HBV incidence may be higher than in urban communities.

## Introduction

First launched in 2007, World Hepatitis Day (WHD) is commemorated annually on 28th July with the aim of uniting the world under one theme to increase awareness and political commitment to tackle the global burden of viral hepatitis [1, 2]. The theme for 2021 was “Hepatitis can’t wait”, highlighting the urgent need to eliminate hepatitis as a public health threat by 2030 amid other health concerns including the ongoing COVID-19 pandemic [3].

The estimated prevalence rates of Hepatitis B virus (HBV) in West Africa range between 6% and 16% in the adult population, and according to reviews published in 2016 [4, 5] Ghana has a national prevalence of about 12.3%. There is however a dearth of information on the vaccination rate for HBV in Ghana. A cross-sectional survey on HBV vaccination status among university students in Ghana established a 38.2% vaccination rate [6].

This high prevalence is reflective of the high burden of infection, along with a high prevalence of chronic liver disease and liver cancer (hepatocellular carcinoma). Hepatitis B and C are the leading causes of liver cancer in Ghana [7]. The Hepatitis Foundation of Ghana reported in 2017 that there are about 4 million Ghanaians living with hepatitis B or C. Regrettably, the majority are not aware of their infection status, leaving them at risk of severe liver disease in later years [8–10] and the potential to unknowingly spread the infections.

Notwithstanding, with the high global burden of HBV and steady efforts being made to improve treatment and care, many people infected remain unaware of their status until they advance in the disease stage. It is established that most low-income countries have only about 5% of infected persons knowing their status. This makes others vulnerable as their ignorance makes them prone to transmit the infection to others around them [11, 12]. This brings up the necessity for more subsidized HBV and HCV screening with better policies and testing standards at health facilities [12, 13] and for exploring the use of other hepatitis awareness-creating approaches that target groups and communities.

In Ghana, HBV is regarded as one of the infections of public health significance and hence warrants matching attention [7]. A safe and effective vaccine that offers 98–100% protection against hepatitis B is available. Vaccination programmes were first introduced and have been available since 1992, 11 years after the first vaccine was approved for use in the USA. They come in the form of monovalent formulations for birth doses or for vaccination of at-risk adult populations like health workers, though not for free [11, 14]. The standard regimen is 3 doses; the second given a month after the first dose, and the third given 6 months after the first dose [15, 16]. Vaccinations for HBV is a key recommendation to control the infection and has proven to be very efficient in conferring immunity [17, 18]. Hitherto HBV and HCV infections can be prevented, controlled and treated - while HBV can be treated, HCV is even curable. This notwithstanding, over 90% of people living with hepatitis B and C in Africa still do not have access to the needed care [19, 20].

A planned awareness and screening exercise was scheduled to coincide with the World Hepatitis Day (WHD) by a University of Ghana-based Hepatitis-Malaria (HEPMAL) project which is funded by the European and Developing Countries Clinical Trials Partnership (EDCTP) and has an objective to investigate the clinical and immuno-pathological consequences of chronic HBV and *Plasmodium falciparum* co-infections [21, 22]. The research group collaborated with recognized bodies and institutions including the Ghana Health Service, Hepatitis Society of Ghana (HEPSoG), Ghana Association for the Study of Liver and Digestive Diseases (GASLIDD), the Hepatitis Foundation of Ghana as well as funding partners such as AngloGold Ashanti to set up various activities to commemorate WHD 2021. Among the prearranged activities was a medical outreach programme which entails education on viral hepatitis, free screening for HBV, as well as free HBV vaccination for screened and eligible individuals.

## Methods

The University of Ghana campus has a student population of about 38,000 when school is in session and close to 5000 members of the community belonging to the staff, their families and others plying their trade within the main campus. Within this community is a sprawling market located centrally around a collection of banks in an area called the ‘banking square’. It is in this area that we mounted three 10 × 10 compact 40-inch canvas canopy tents for our walk-in service. We set up to provide to interested members of the community screening and vaccinating for HBV at no cost to them. They were first taken through a registration process where details including name, contact numbers and previous vaccination history were taken. The brand of vaccines we administered were Engerix-B (Hep-B) - noninfectious recombinant DNA vaccine containing hepatitis B surface antigen (HBsAg). It is produced by Glaxo Smith Kline (GSK), UK, from genetically engineered yeast (Saccharomyces cerevisiae).

Clinicians and nurses from the University of Ghana Hospital (UGH), the University of Ghana Medical Centre (UGMC), the Korle-Bu Teaching hospital (KBTH) and the Greater Accra Regional Hospital were in attendance to provide education on viral hepatitis, pre- and post-vaccination counselling services, and to administer the vaccines to eligible individuals. Registered individuals were given a brief background of the purpose of the event and educated on viral hepatitis including the mode of transmission, types and stages of the disease condition including complications resulting from the infection. Eligible volunteers who consented were then taken through a pre-test counselling session before 2 ml of venous blood was collected into a sterile EDTA vacutainer tube with a single use butterfly needle blood collection kit. Eligible volunteers are made to self-report during the verbal counselling session on their awareness, knowledge levels and vaccination status of HBV.

Haemoglobin levels were determined using a portable haemoglobin meter (URIT-12 Haemoglobin Meter, Accurex Biomedical Pvt. Ltd, Mumbai, India) with the recommended test strips. A Ministry of Health (MoH) / Ghana Health Service (GHS) recommended HBV rapid test strip, ‘The Advanced Quality One-Step multi-HBV test kit’ (InTec Products Inc, Xiamen, PRC) was used to determine the HBV status of the participants. The test panel was used to determine the HBsAg, HBsAb, HBeAg, HBcAg and HBeAb status of the participants. Those that tested negative for both HBsAg and HBsAb all accepted and were vaccinated, while those that tested negative for HBsAg but positive for HBsAb were not vaccinated since they already had antibodies against the virus. A rigorous follow-up schedule of making telephone calls a week before and on-the-day reminders to all vaccinees for the second and the third doses was prearranged. Those that tested positive for only HBsAg were taken through a post counselling session and were booked for hospital appointments at the UGH. The medical screening and vaccination exercise which was captured under the activities of the HEPMAL project had an Institutional Review Board (IRB) approval from the NMIMR IRB with the number, NMIMR-IRB CPN 046/19–20.

## Results

In all, 297 participants between the ages of 17 and 67 years attended the event, 126 (42%) of whom were males and 171 (58%) were females (Table 1). Of this number, 283 (Table 2) were screened, amongst which 19 (6.4%) tested positive for HBsAg, all of whom were also positive for HBeAg and HBcAb. Additionally, 17 (5.7%) tested positive for HBsAb, out of which one also tested positive for HBcAb. From all the participants screened, only 1 person tested positive for HBeAb and negative for all other antigens and antibodies. Twenty-one (7.1%) of the participants tested positive for HBcAb, two of whom were negative for HBsAg, HBeAg and HBsAb; only one was positive for HBsAb.

A total number of 59 (19.9%) of the eligible volunteers had previously taken the HBV vaccination. Three of the 59 tested positive for HBV surface antigen. The first of the 3 doses of the HBV vaccine were successfully administered to 246 of participants of which 131 were males (Table 2) who tested negative for all HBV antigens and antibodies. By the end August 2021, which was the due date to complete vaccination for the second doses, 196 (79.7%) of the 246 who took the first dose had taken their second dose. This meant 50 (20.3%) individuals were lost to follow-up. The final dose which was administered six months (January 2022) after the first dose registered 163 vaccinees out of the 196 eligible recipients with 33 (16.8%) individuals lost to follow-up. Vaccine success rate overall was therefore 66%.

**Table 1:** Results summary

Category			Results		
			Male	Female	Total
**Number of participants**	**Gender**		126	171	297
	**Mean Age (Range)**		31.5 (19–67)	28.4 (17–64)	
**Vaccination history**	Vaccinated*		18	41	59
	**Not vaccinated**		107	129	236
	**Unspecified** ^**#**^				2
**HBV Profile**	**HBsAg**	Positive	14	5	19
		Negative	108	156	264
	**HBsAb**	Positive	6	11	17
		Negative	116	150	266
	**HBeAb**	Positive	0	1	1
		Negative	122	160	282
	**HBeAg**	Positive	14	5	19
		Negative	108	156	264
	**HBcAb**	Positive	15	6	21
		Negative	107	155	262

*Shows people who have previously initiated vaccination, i.e., taken at least 1 dose of the vaccine prior to this screening exercise

^#^Did not say whether or not they have received HBV vaccination prior to this exercise

Table 2: **HBV Vaccine administration at medical outreach –** (a) Medical outreach participants and vaccine recipients: a breakdown of number of participants who attended the medical outreach, those successfully screened and those eligible for vaccination as well as eligible vaccine recipients who successfully received first, second and third doses of HBV vaccines – others lost to follow-up.

**Table 2:** Vaccination summary

Category			Results		
			Male	Female	Total
**Age (yrs.)**			19–67	17–64	
**Mean Age (yrs.)**			31.5	28.4	
**Vaccination status** ^*^	**Previously vaccinated**		18	41	59
	**Unvaccinated**		107	129	236
**HBV Profile**	**HBsAg**	Positive	14	5	19
		Negative	108	156	264
	**HBsAb**	Positive	6	11	17
		Negative	116	150	266
	**HBeAb**	Positive	0	1	1
		Negative	122	160	282
	**HBeAg**	Positive	14	5	19
		Negative	108	156	264
	**HBcAb**	Positive	15	6	21
		Negative	107	155	262

*Shows people who have taken at least 1 dose of the HBV vaccine prior to this screening exercise

## Discussion

The WHO’s Global Health Sector Strategy (GHSS) on viral hepatitis hopes to achieve elimination of viral hepatitis by 2030. This means reducing the annual disease incidence and mortality by 90% and 65% respectively using 2015 data as baseline [7]. Diagnosis and awareness of infection is part of the first essential steps towards achieving this goal. The HEPMAL Project which is an EDCTP2-funded study on the immunopathological effects of HBV and malaria co-infections, sought to address and support this drive with an HBV educational, screening and vaccination exercise on the University of Ghana’s main campus. Our screening and vaccination exercise examined the awareness rate among the volunteers and the prevalence of HBV within the University community. We established from the eligible volunteers a suboptimal rate in awareness through our verbal counselling sessions and an active case prevalence of 6.4%. This suggests an ongoing active viral replication and individuals in this category who have the HBV have an increased risk of liver cancer if they remain unaware of their infection. This prevalence rate is comparable to the estimated 8.36% national prevalence for adults in a systematic review and meta-analysis of HBV infections between 2015 and 2019 in Ghana [23]. The same study reported prevalence rates of 0.55% and 14.30% among pre-school children and adolescents, respectively [23].

In Ghana, HBV is the most dominant cause of viral hepatitis and requires immediate attention, especially through rapid implementation of the birth dose HBV vaccines, active case search for timely intervention, and vaccination of uninfected adults^24^. We used Engerix-B HBV vaccines for our exercise. This is a 3-dose series with 2 doses separated by ≥ 4 weeks, and a 3rd dose 4 to 6 months after the 2nd dose. An individual lost to follow-up for not completing the series, for example missing out on the second and/or the third dose, the series does not need to be restarted. Though we had a pre-counseling session with the needed information on the vaccines including the dose-scheduling for all vaccinees, our vaccination coverage for all negative anti-HBV antibody volunteers was not completed. We lost 50 (20.3%) and 33 (16.8%) persons to follow-up for the second and third dose series respectively. Through the rigorous call up and reminder schedules we set up for vaccinees prior to their return for the second and the third dose series, we had various reasons including reallocation and simply making time to come over for the dose. Overall, we achieved a full vaccination success rate of about 66%, largely due to the follow-up strategy that was implemented.

Data gathered through self-reports indicated that 19% of participants had previously had HBV vaccines but indicated they are yet to complete the three-dose regimen. Though relatively an easy means to obtain data, self-reports are limited by reliability and validity. These participants who are yet to complete their three-dose regimen took at least one dose of the vaccine more than 6 months prior to this screening. Three out of this number had since become infected with the virus, testing positive for HBsAg alone. These are likely to have been infected before they took their first shot. They have not developed protective antibodies, and this may be attributed to their inability to complete the full series of the vaccines. Eligible volunteers who passed for vaccination during our event were advised of the importance of completing the series of the vaccine regimen. Even with the counseling, 20% of the of the number that took the first dose of the vaccine did not show up for the second dose. This may be attributed to various reasons including genuine inability to be present due to residency but may not be hesitancy for the vaccines. Control programmes may need to explore home or school vaccination strategies as is implemented for the polio vaccine in developing countries to minimize losses to follow-up.

Another interesting observation is the finding that a higher number of males (60) were lost to follow up than females (23), even though more males signed up for vaccination at the beginning. This may be important for informing gender-oriented follow-up strategies that will improve upon full vaccine uptake.

The Ghana Health Service (GHS) is on the verge of implementing HBV birth dose in health facilities nationwide, which can be complemented with active surveillance activities as opposed to the current passive surveillance method employed to accelerate HBV elimination. This will identify infected people who may be living apparently healthy lives for timely intervention to increase their life expectancy and curb community transmission. Additionally, we recommend private sector partnerships to assist in funding such laudable endeavors and extending them beyond WHD commemoration events. Our findings also emphasize the need to increase awareness creation for viral hepatitis in Ghanaian communities even among the literate to encourage voluntary vaccinations among teenagers and adults who were born before the HBV birth dose was implemented. We encourage more of such programmes and hope to partner with other corporate institutions to extend this to rural communities as well.

Limitations included the total number (sample size) and the conflict of biased representativeness of sampled eligible participants within the university community. Considerations of the use of larger numbers and a good representative community as well as the use of structured questionnaires for appropriate meta data from eligible participants are planned for subsequent exercises.

The use of screening strategies in communities to target groups is expected to be vital to the goals for general awareness-raising. Assessment of the effectiveness of this many-sided approach is necessary to reduce the undiagnosed population and improve the link between testing and care for viral hepatitis.

## Data Availability

All data generated or analyzed during this study are included in this published article.
